# A novel signature incorporating lipid metabolism- and immune-related genes to predict the prognosis and immune landscape in hepatocellular carcinoma

**DOI:** 10.3389/fonc.2023.1182434

**Published:** 2023-06-06

**Authors:** Ti Yang, Yurong Luo, Junhao Liu, Fang Liu, Zengxin Ma, Gai Liu, Hailiang LI, Jianfan Wen, Chengcong Chen, Xiancheng Zeng

**Affiliations:** ^1^ Department of Hepatobiliary-Pancreatic and Hernia Surgery, Guangdong Second Provincial General Hospital, Guangzhou, China; ^2^ The Second School of Clinical Medicine, Southern Medical University, Guangzhou, China; ^3^ The First School of Clinical Medicine, Southern Medical University, Guangzhou, China; ^4^ Department of Radiation Oncology, Affiliated Cancer Hospital and Institute of Guangzhou Medical University, Guangzhou, China

**Keywords:** liver hepatocellular carcinoma, prognostic gene signature, lipid metabolism, tumor immune microenvironment, survival prediction, individualized chemotherapy, targeted chemotherapy

## Abstract

**Background:**

Liver hepatocellular carcinoma (LIHC) is a highly malignant tumor with high metastasis and recurrence rates. Due to the relation between lipid metabolism and the tumor immune microenvironment is constantly being elucidated, this work is carried out to produce a new prognostic gene signature that incorporates immune profiles and lipid metabolism of LIHC patients.

**Methods:**

We used the “DEseq2” R package and the “Venn” R package to identify differentially expressed genes related to lipid metabolism (LRDGs) in LIHC. Additionally, we performed unsupervised clustering of LIHC patients based on LRDGs to identify their subgroups and immuno-infiltration and Gene Ontology (GO) enrichment analysis on the subgroups. Next, we employed multivariate, LASSO and univariate Cox regression analyses to determine variables and to create a prognostic profile on the basis of immune- and lipid metabolism-related differential genes (IRDGs and LRDGs). We separated patients into low- and high-risk groups in accordance with the best cut-off value of risk score. We conducted Decision Curve Analysis (DCA), Receiver Operating Characteristic curve analysis as a function of time as well as Survival Analysis to evaluate this signature’s prognostic value. We incorporated the clinical characteristics of patients into the risk model to obtain a nomogram prognostic model. GEO14520 and ICGC-LIRI JP datasets were employed to externally confirm the accuracy and robustness of signature. The gene set variation analysis (GSVA) and gene set enrichment analysis (GSEA) were applied for investigating the underlying mechanisms. Immune infiltration analysis was implemented to examine the differences in immune between both risk groups. Single-cell RNA sequencing (scRNA-SEQ) was utilized to characterize the genes that were involved in the distribution of signature and expression characteristics of different LIHC cell types. The patients’ sensitivity in both risk groups to commonly used chemotherapeutic agents and semi-inhibitory concentrations (IC50) of the drugs was assessed using the GDSC database. On the basis of the differentially expressed genes (DEGs) in the two groups, the CMAP database was adopted for the prediction of potential small-molecule compounds. Small-molecule compounds were molecularly docked with prognostic markers. Lastly, we investigated the prognostic gene expression levels in normal and LIHC tissues with immunohistochemistry (IHC) and quantitative reverse transcription polymerase chain reaction(qRT-PCR).

**Results:**

We built and verified a prognostic signature with seven genes that incorporated immune profiles and lipid metabolism. Patients were classified as low- and high-risk groups depending on their prognostic profiles. The overall survival (OS) was markedly lower in the high-risk group as compared to low-risk group. Time-dependent ROC curves more precisely predicted patients' survival at 1, 3 and 5 years; the area under the ROC curve was 0.81 (1 year), 0.75 (3 years) and 0.77 (5 years). The DCA curves showed the value of the prognostic genes in this signature for clinical applications. We included the patients' clinical characteristics in the risk model for both multivariate and univariate Cox regression analyses, and the findings revealed that the risk model represents an independent factor that influences OS in LIHC patients. With immune analysis, GSVA and GSEA, we identified that there are remarkable differences between the two risk groups in immune pathways, lipid metabolism, tumor development, immune cell infiltration and immune microenvironment, response to immunotherapy, and sensitivity to chemotherapy. Moreover, those with higher risk scores presented greater sensitivity to the chemotherapeutic agents. Experiments *in vitro* further elucidated the roles of SPP1 and FLT3 in the LIHC immune microenvironment. Furthermore, four small-molecule drugs that could target LIHC were screened. *In vitro* qRT-PCR , IHC revealed that the SPP1,KIF18A expressions were raised in LIHC in tumor samples, whereas FLT3,SOCS2 showed the opposite trend.

**Conclusions:**

We developed and verified a new signature comprising immune- and lipid metabolism-associated markers and to assess the prognosis and the immune status of LIHC patients. This signature can be applied to survival prediction, individualized chemotherapy, and immunotherapeutic guidance for patients with liver cancer. This study also provides potential targeted therapeutics and novel ideas for the immune evasion and progression of LIHC.

## 1 Introduction

Liver hepatocellular carcinoma (LIHC) is the sixth most frequent cancer and the fourth major cause of death associated with cancer globally, with 782,000 deaths and 841,000 new cases each year. By 2030, more than one million patients are predicted to die of liver cancer (1). LIHC is the most frequent histological form of liver cancer and has a poor prognosis (2). Except for individuals diagnosed early or suitable for potentially curative treatment, the therapy for advanced LIHC is limited by its heterogeneity, and the overall prognosis of patients with LIHC remains unsatisfactory (3, 4). The overall survival (OS) associated with LIHC treatment also remains unsatisfactory. The earlier the diagnosis of primary LIHC, the greater the treatment success rate, which is important for improving the quality of prognosis. Hence, more reliable and sensitive prognostic indicators are urgently required to monitor the progress of LIHC and assess the survival of patients.

Recently, metabolic reprogramming has been recognized as a hallmark of malignancy (5). The contribution of lipid metabolism in the cancer context has attracted growing attention, and the multiple roles of lipids in energy sources, membrane composition and cell signaling are vital for cancer cell (6). In addition, several chemical inhibitors of fatty acid biosynthetic pathways inhibit the development of LIHC: HDAC3 inhibitors destabilize FASN proteins and inhibit the growth of LIHC (7). The combination of targeted SCD and sorafenib may have a synergistic effect on LIHC (8), and a recently developed organic small molecule, fluoxetine, inhibits the endoplasmic reticulum (ER) of SREBP-1 by binding to the SCAP-Golgi translocation complex, thereby strongly inhibiting SREBP-1 activation to suppress LIHC development (9). Consequently, modulation of lipid metabolism has been determined as an underling therapeutic target to enhance the prognosis of LIHC patients. Besides, several researches have tried to develop prognostic models for LIHC patients on the basis of genes related to lipid metabolism (10–12). However, the robustness and effectiveness of single-feature models are relatively poor; therefore, insights into multi-feature signaling models and their prognostic impact in patients with LIHC are required.

**Figure 1:**
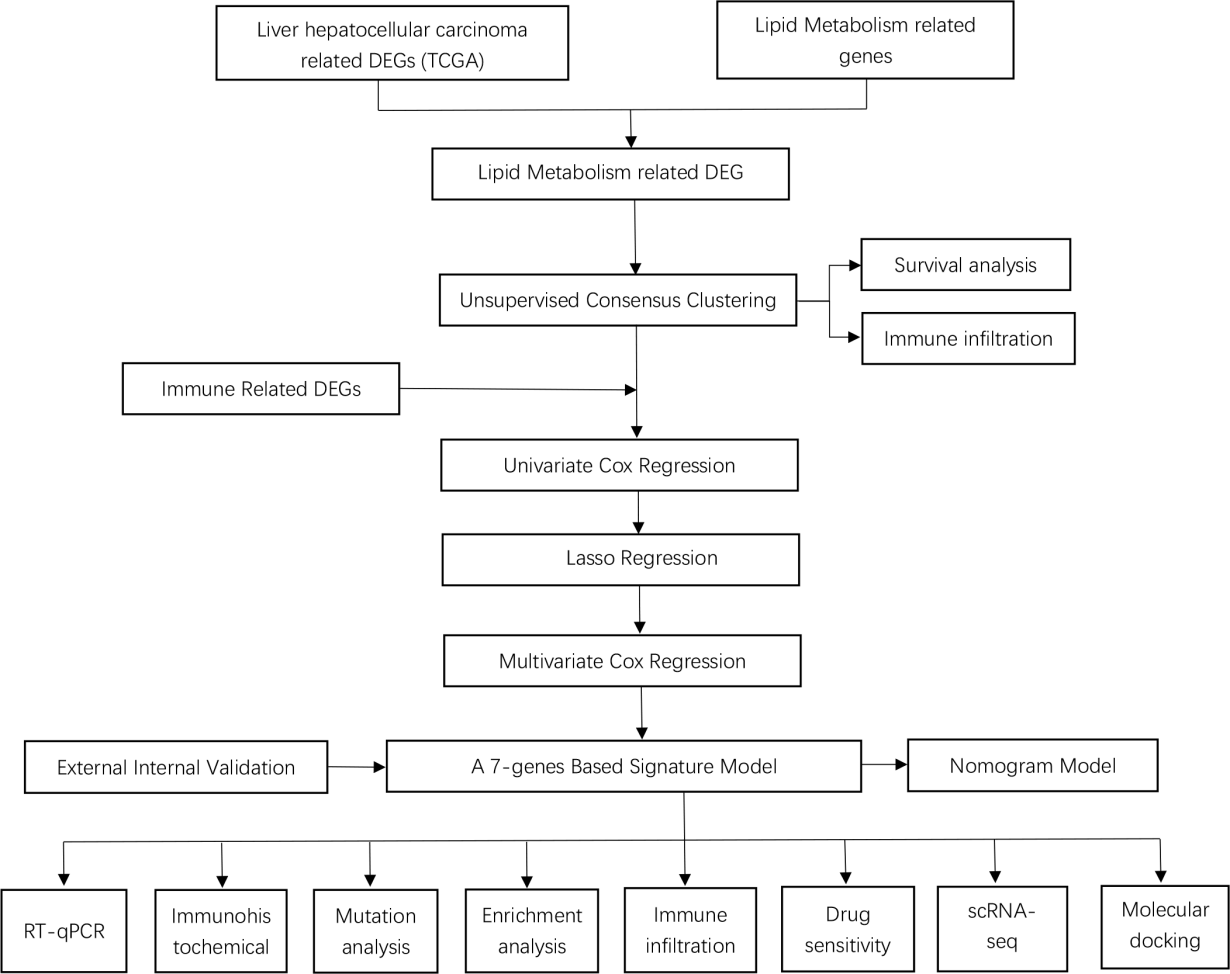
The flow diagram of the present study.

Recent researches have suggested that the reprogramming of lipid metabolism is not restricted to tumor cells, but is also strongly linked to the function of the immune cells that permeate the tumor microenvironment. As an example, it has been indicated that lipid oxidative phosphorylation and elevated lipid uptake are necessary for the polarization of tumor-associated macrophages, and CD36, the lipid uptake-associated molecule is determined to be a promising tumor marker (13). Similarly, since NKT cells predominantly identify lipid antigens, alterations in the metabolic status of tumor lipids can alter the lipid antigen pool, which may influence their immunomodulatory function (14). Moreover, Dendritic cells (DCs) from patients with LIHC have an impaired ability to trigger immune responses while promoting immunosuppression. The regulation of lipid metabolism, such as slave FAS during the activation of DCs, affects the endoplasmic reticulum (ER) and Golgi amplification and thus their antigen-presenting capacity (15). Besides, we sought to build a new model for the prediction of prognosis in LIHC patients by incorporating genes associated with immune and lipid metabolism, depending on the interactions between anti-tumor immunity and lipid metabolism. This study aimed to identify LIHC patient subgroups on the basis of various lipid metabolism characteristics, an unsupervised clustering method based on TCGA-CIHC was applied. Comparisons were made between subgroups for differences in the levels of immune infiltration and OS. Least absolute shrinkage with selection operator (LASSO) regression and Cox regression were employed to incorporate immune- and lipid metabolism-related DEGs into the model. Screening was performed using GEO14520 and ICGC-LIRI JP for external verification. We carried out somatic mutation and functional enrichment analyses to investigate potential mechanisms for differences in survival between risk groups. Ultimately, the relation between the risk scores and sensitivity to chemotherapeutic agents and the immune infiltration levels was assessed. Risk models were analyzed at the single-cell level, and drug prediction and molecular docking were performed for risk-associated genes. Prognostic gene expression in normal and LIHC samples was verified *via* qRT-PCR and IHC. An overview of the flowchart of this study is presented in **Figure 1**.

## 2 Materials and methods

### 2.1 Data collection and preprocessing

The RNA sequencing data, mutation profiles and clinical information of LIHC patients were downloaded from the TCGA data portal. DEGs were determined through differential analysis of original RNA sequencing data. All of the transcriptome data were transformed logarithmically and transformed to transcripts per million (TPM) before analysis. For the external verification, GSE14520 clinical information together with RNA-sequencing data were acquired from the GEO database. The ICGC-LIRI JP clinical information and RNA-sequencing data were derived from the ICGC. Additionally, 34 gene sets associated with lipid metabolism were acquired from the Molecular Signature Database. These were combined to obtain 1996 lipid metabolism-associated genes (**Supplementary Table 1**). In addition, we downloaded 2499 genes associated with immune (**Supplementary Table 2**) from the ImmPort database. After removing duplicate genes, we obtained 1811 immune-related genes.

### 2.2 Identification of DEGs linked to lipid metabolism and immunity

A differential gene expression analysis of normal and LIHC tissues from the TCGA database was carried out with the 'DEseq2' R package. DEGs with an adjusted P value<0.05 and an absolute fold change (|logFC|) >1 was chosen. We subsequently crossed immune- and lipid metabolism-associated genes with DEGs. In total, 522 LRDGs and 395 IRDGs were determined in follow-up analyses. Detailed information on immune- and lipid metabolism-associated genes is provided in **Supplementary Table 3**.

### 2.3 Unsupervised consensus clustering of LRDGs and the analysis of their immune status

Subgroups of patients with LIHC on the basis of LRDGs were determined with 'ConsensusClusterPlus'R package. For ensuring the reproducibility of our method, we set an arbitrary random seed number of 99 in the 'ConsensusClusterPlus' software package and obtained the best number of clusters as 2 according to cumulative distribution function (CDF) curves, cluster consistency, as well as the relative variation of area under the CDF curve to profile clustering indices. Each of the survival curves of clusters was analyzed with Kaplan-Meier method. The immune cell infiltration pattern between normal tissue and tumor microenvironment in LIHC was examined using several immune correlation algorithms such as ssGSEA and CIBERSORT. We also performed the same analysis for lipid metabolism subtypes.

### 2.4 Construction and validation of a prognostic signature based on both LRDGs and IRDGs

Since there is a strong link between the patterns of immune infiltration and lipid metabolism, we attempted to build a prognostic profile combining IRDGs and LRDGs to evaluate the prognosis of LIHC patients and verify its feasibility. Firstly, the prognostic genes on the basis of 395 IRDGs and 522 LRDGs were chosen in the TCGA dataset *via* univariate Cox regression ('survival' R package). Candidate genes were subsequently screened with LASSO regression ('GLMNET'R package). Lastly, the candidate genes were subjected to multivariate Cox regression ('survival' R package) and prognostic features were built. By multiplying a linear combination of expression levels of gene with the multivariate Cox regression coefficient (β), a risk score was calculated. The detailed formula for the risk score was identified as below:


$$\;\;\;\;\;\;Risk\;Score=\sum_{i=1}^{n}Coefficient\left(\beta_{i}\right)*x_{i}$$

In this formula, $x_{i}\;$ stands for the level of expression of prognostic signature gene and $Coefficient\left(\beta_{i}\right)\;$ for the respective regression coefficient.

In accordance with the median risk score, LIHC patients could be categorized into low- and high-risk groups. Clinical decision analysis (DCA), time-dependent ROC curves and survival curves were also performed on the constructed models, and the curves were plotted to assess their clinical benefits. For external validation, the reliability and accuracy of the seven-gene models were verified using GSE14520 and ICGC-LIRI JP.

### 2.5 Building and evaluating a predictive nomogram model for patients with LIHC

We incorporated clinical features into a seven-gene risk model and conducted multivariate and univariate Cox regression analyses for each factor. In accordance with the outcomes of multivariate regression, we established a nomogram model for prognostic evaluation through integrating tumor TNM staging into the prognostic labels of seven genes. Based on the prognostic model calibration curves were drawn to identify the predictive reliability of model at 1.3.5 years. DCA was employed for assessing the net clinical benefit of the prognostic model.

### 2.6 Functional annotation and enrichment analysis

We combined IRDGs and LRDGs for Kyoto Encyclopedia of Genes and Genomes (KEGG) and gene ontology (GO) enrichment analysis ('clusterProfiler' R package) to investigate pathways that may be enriched for tumor development (**Supplementary Figure 2**). In the Molecular Signatures Database (16), based on the definition of C2 (C2.cp. Kegg.v7.4.symbols.gmt) retrieved from the database, a GSEA was conducted to evaluate underlying differences in biological function between the risk groups. For GSEA, the entries with false discovery rates<0.25 and corrected P-values<0.05 were deemed significant. A genomic variance analysis (GSVA) algorithm was also employed to determine the signaling pathways enriched among both risk groups ('GSVA' R package) based on the 50 tagged signaling pathways highlighted in the molecular signature database (17).

### 2.7 Tumor immune infiltration analysis

To evaluate the abundance of diverse immune cell infiltrates in tumor tissues of both risk patients, the tumor immune microenvironment was also examined with CIBERSORT algorithm. We calculated the immune fraction, mesenchymal fraction, tumor purity and estimated fraction for each patient with LIHC with the application of an estimation algorithm. Dysfunction, TIDE, as well as exclusion scores were derived from the Tumor Immune Dysfunction and Exclusion website (18) to evaluate the capacity of immune escape together with response to immunotherapy in both risk group. The tumor immunophenotyping (TIP) website is a single-stop platform for rapidly analyzing and visualizing the immune activity that kills tumors (also known as the cancer immune phase). We compared the different phases of the anti-tumor immune responses in both risk patients using the TIP website (**Supplementary Figure 3**).

### 2.8 Somatic cell mutation analysis

In view of the somatic mutation models in the TCGA database, the 'MATFOOL' R package was utilized to plot waterfalls to visualize the somatic mutation frequencies and the distribution of various types of mutated genes in low- and high-risk patients.

### 2.9 scRNA-seq analysis

Tumor Immunological Single Cell Centre (TISCH) database includes 79 high-quality single-cell transcriptomic datasets from 27 tumors in the ArrayExpress and GEO databases, together with the appropriate clinical information, giving detailed cell type annotation at the single-cell level. The database has the benefits of ease of use, data completeness, data visualization and usability (19). The expression and distribution of signature genes in the GSE166635 dataset were visualized from the TISCH database using Unified Streaming Approximate Projection (UMAP) plots.

### 2.10 Chemotherapy response and small molecule drug screening

Genomics of Drug Sensitivity in Cancer database is a publicly available genomics database of antitumor drug sensitivity devoted to determining molecular markers of cancer and predicting the target responses to antitumor drug (20). We predicted the susceptibility of LIHC patients to nine commonly used chemotherapeutic agents from the GDSC database. The chemotherapeutic drug response was calculated in LIHC patients *via* applying the 'pRRophetic' R package. The Connectivity Map Database (CMap) is a biologic database that uncovers the functional connections between disease states, genes and small molecule compounds (21, 22). DEGs that were down- and up-regulated in both risk groups were downloaded to the CMAP database for predicting the small-molecule drugs that could be utilized for the treatment of LIHC (p<0.05), revealing the relationship among small-molecule compound function, genes, and disease state. In addition, we utilized the PubChem-accessible chemical database, which gives the three-dimensional structures of small-molecule drugs.

### 2.11 Molecular docking analysis

We selected prognostic genes with hazard ratios greater than 1 as the target genes. Sequences along with the annotation information for proteins were acquired from the Universal Protein Resource database. Protein structures of the key targets (SPP1, STC2, GAL, and KIF18A) were downloaded from the Protein Data Bank (http://www.rcsb.org,pdb). AutoDock Tools software (version 1.5.6) was used to molecularly dock the key targets for small-molecule drugs. PyMOL software was employed for removing the small-molecule ligands and water molecules, evaluating their binding activity according to docking energy values and finally visualizing the docking results.

### 2.12 Human specimens

Human specimens were taken from LIHC patients who were admitted to the Department of Hepatobiliary-pancreatic&hernia surgery, Guangdong Second People's Hospital. Seven pairs of LIHC and paracancerous specimens were collected. This work was granted approval by the Medical Research Ethics Committee of Guangdong Second People's Hospital. All of the patients in this work were given written informed consent. After specimen isolation, liver tissue was frozen rapidly in the liquid nitrogen and was stored in a refrigerator at -80°C to prevent degradation.

### 2.13 qRT-PCR

Total RNA were isolated using Trizol reagent (Invitrogen, Carlsbad, CA, USA). cDNA was amplified using ABI 7500 Fast System (Applied Biosystems, Rockville, MD, USA). The gene of α-Tubulin is used as the reference. The relative expression of genes was calculated by the equation: 2-[(Ct of gene)-(Ct of α-tubulin)], and the Ct represents threshold cycle. The primers are as follows: FLT3, forward 5’- GCCGCTGCTCGTTGTTTT-3’ and reverse 5’-ACACACTTGATCACAGGCAGA-3’; SPP1, forward 5’-AAGCAGCTTTACAACAAATACCCAG-3’ and reverse 5’- TGGACTTACTTGGAAGGGTCTGTG-3’; KIF18A, forward 5’-TGCTGGGAAGACCCACACTAT-3’ and reverse 5’-GCTGGTGTAAAGTAAGTCCATGA3’; SOCS2, forward 5’-TTAAAAGAGGCACCAGAAGGAAC-3’ and reverse 5’-AGTCGATCAGATGAACCACACT-3’.

### 2.14 Immunocytochemistry

We detected protein expression by IHC experiments for SPP1, FLT3, KIF18A, SOCS2. Fresh human tissues were taken and fixed with 10% formalin overnight, dehydrated, paraffin embedded, sectioned, dewaxed, hydrated, antigen repaired with citrate, peroxidase blocked in liver by 3% H_2_O_2_, the primary antibodies SPP1 (1:100, T55333S, Abmart), FLT3 (1:500, T611358S, Abmart), KIF18A (1:50, PK51852S, Abmart), SOCS2 (1:50, R25765, Zen BioScience) were incubated at 4°C overnight. Then, the secondary antibody (1:500, 511203 , Zen BioScience) was incubated for 1 hour at 374°C, DAB color development kit, hematoxylin re-stained, and finally, dehydrated and transparent, neutral treacle sealed. And observed under a microscope, two experienced pathologists performed double-blind readings and scored the percentage of positive cells and staining intensity, respectively. The percentage of positive cells was scored as follows: (5%, 0 points; 5%-25%, 1 point; 26%50%, 2 points; 51%-75%, 3 points; 76%-100%, 4 points. Staining intensity was evaluated according to the following criteria: 0 as colorless; 1 point for light yellow; 2 points for tanning; and 3 points for Brown team. The percentage of positive cells and staining intensity were multiplied to obtain the final score. Among them, 0 was scored as negative (–); weakly positive (+) 1-4, positive (++) 5-8, and strongly positive (++++) 9-12.

### 2.15 Statistical analysis

R software (version 4.2.0, https://www.r-project.org/) and the related R package were employed to analyze and visualize the data. Comparisons among both groups were made with the Wilcoxon rank-sum test, and comparisons between two or more groups were implemented through the Kruskal-Wallis test. Comparisons of categorical variables were conducted utilizing the chi-square test or Fisher's exact test. The differences between survival curves were identified *via* applying the log-rank test. Associations between both variables were evaluated with Spearman's correlation test. Statistical significance was set to P<0.05. significant

## 3 Results

### 3.1 Identification and exploration of DEGs related to lipid metabolism and immunity

**Figure 2:**
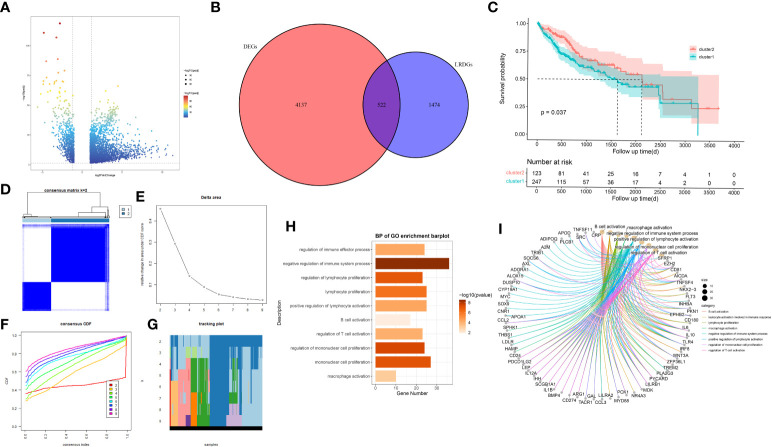
Exploration of lipid metabolism-related DEGs (LRDGs). **(A)** TCGA-LIHC volcano map of differentially expressed genes. **(B)** The Venn diagram displays the intersection of common genes among LIHC-related DEGs and lipid metabolism-related genes. **(C)** Kaplan-Meier curves for overall survival of LIHC patients in different clusters. **(D)** unsupervised consensus clustering heatmap. **(E)** The plot of the relative area changes from k=2 to 9 under the cumulative distribution function (CDF) curve. **(F)** Consistent CDF plot. **(G)** Tracing plot of clustered samples. **(H, I)** A bar plot **(H)** and chord diagram **(I)** showing immune-related biological processes enriched to LRDGs by GO analysis.

We utilized TCGA-CIHC to identify genes that were differentially expressed between the normal and tumor tissues. We gained 4659 DEGs with criteria of (|logFC|)>1 and P<0.05 (**Figure 2A**). Additionally, these genes intersect with genes associated with lipid metabolism (**Figure 2B**), resulting in 522 LRDGs (**Supplementary Table 3**).

Unsupervised consistency clustering analysis was conducted for LIHC patients on the basis of LRDGs expression to obtain two lipid metabolism subgroups (**Figures 2D-G**). Survival analysis of the two groups revealed a considerable difference in the survival time between both groups (**Figure 2C**). Similar clustering patterns were observed in the GSE14520 dataset (**Supplementary Figure 1**). We performed GO analysis of LRDGs, and the biological processes indicated that LRDGs were markedly enriched in modulating the processes of immune effects, lymphocyte proliferation, B-cell activation, T-cell activation, monocyte proliferation, and macrophage activation (**Figures 2H, I**).

**Figure 3:**
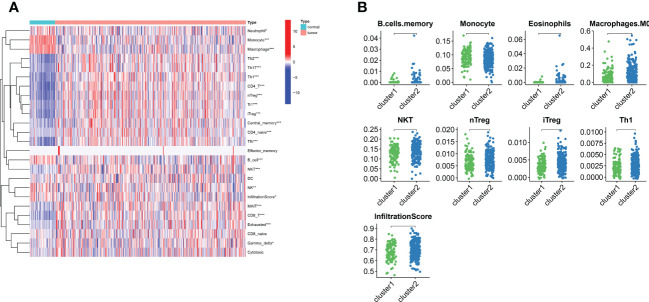
The landscape of LIHC immunity. **(A)** Comparison of immune cell infiltration patterns between tumor tissue and normal tissue by ssGSEA algorithm. **(B)** Comparison of immune cell infiltration patterns between different clusters. *p < 0.05, **p < 0.01, and ***p < 0.001.

For examining the underlying reasons of survival differences, the immune infiltration of normal and tumor tissues in LIHC was analyzed and heat maps were drawn. In LIHC, there existed obvious differences in the degree of immune infiltration between normal and tumor tissues. In particular, regulatory T cells, mononuclear macrophages, CD8+ T cells, helper T cells and CD4+ T cells were observed elevated in cluster 2 (**Figure 3A**). Immune infiltration analysis of the two different lipid metabolism patterns revealed significant differences between monocytes, macrophages, regulatory T cells and helper type I T cells (**Figure 3B**). Together, these analyses suggest that the patterns of lipid metabolism are strongly associated with immune infiltration as well as prognosis in LIHC patients, indicating the combination of immune- with lipid metabolism-associated genes to develop the clinical prognostic models is an ideal approach.

### 3.2 Enrichment analysis

Functional enrichment analysis of IRDGs and LRDGs revealed KEGG (**Supplementary Figures 2B–D**) enrichment in alcoholic liver disease, hepatitis B, cytokine-cytokine interactions, fat digestion and absorption, choline metabolism in cancer, PPAR signaling pathway, IL-17 signaling pathway, the interaction of viral proteins with cytokines as well as cytokine receptors, and natural killer cell-mediated cytotoxicity. The enriched GO (**Supplementary Figures 2A, B**) molecular functions were ligand-receptor activity, cytokine activity, immune receptor activity, lipase activity, phospholipase activity, and other pathways. Biological processes were enriched at the level of lipid localization, lipid transport, response to negative regulation of the steroid hormone immune system, regulation of molecular mediator production in immune effects, adaptive immune responses based on lymphocyte-mediated immunity, monocyte differentiation, and other pathways. Enriched cellular components included the neuronal cytosol, endoplasmic reticulum lumen, secretory granule lumen, immunoglobulin complex, transcriptional regulatory complex, and cellular matrix.

### 3.3 Construction and validation of a prognostic signature based on both LRDGs and IRDGs

**Figure 4:**
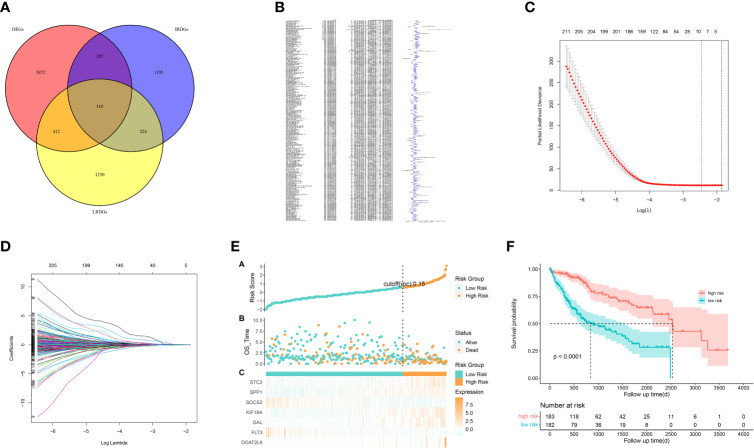
Construction of a prognostic signature for LIHC patients based on immune-related and lipid metabolism-related DEGs. **(A)** The Venn diagram displays the intersection of common genes among LIHC-related DEGs and lipid metabolism-related and immune-related genes. **(B)** The forestplot shows the results of hazard ratios and 95% confidence intervals of signature genes from the univariate Cox regression analysis. **(C)** The LASSO regression algorithm was used to select the optimal variable (l) with a 10-fold cross-validation method. **(D)** The solution path was plotted according to coefficients against the L1 norm. **(E)** The distribution of risk score, survival status, and the expression levels of coefficients in the prognostic signature. **(F)** The overall survival curves of LIHC patients between high-risk and low-risk groups were plotted based on the prognostic signature.

We identified 522 LRDGs and 395 IRDGs by intersecting the genes related to immune signature and lipid metabolism with DEGs, respectively (**Figure 4A**). Univariate Cox regression analysis was implemented on 395 IRDGs and 522 LRDGs to screen 133 candidate genes with prognostic value (**Figure 4B**). LASSO regression (**Figures 4C, D**) and multivariate Cox regression analyses were implemented. DGAT2L6, SOCS2, GAL, FLT3, KIF18A, STC2, and SPP1 were employed as a prognostic indicator to build a risk model with 0.741 C-index. Patients were categorized into low-risk (n = 183) and high-risk (n = 182) groups depending on the median risk score. The patients' baseline features on the basis of risk model are given in **Table 1**.

**Table 1:** Baseline characteristics and comparison of LIHC patients divided by the prognostic model.

characteristics	levels	Low-risk	Low-risk	pvalue	method
	n	183	182		
event, n (%)	Alive	140 (38.4%)	99 (27.1%)	<0.001	Chisq test
	Dead	43 (11.8%)	83 (22.7%)		
Age, n (%)	<65	104 (28.5%)	112 (30.7%)	0.36	Chisq test
	≥65	79 (21.6%)	70 (19.2%)		
Gender, n (%)	FEMALE	58 (15.9%)	61 (16.7%)	0.71	Chisq test
	MALE	125 (34.2%)	121 (33.2%)		
tumor_grade, n (%)	G1	40 (11%)	15 (4.1%)	<0.001	Yates' correction
	G2	97 (26.6%)	78 (21.4%)		
	G3	38 (10.4%)	80 (21.9%)		
	G4	4 (1.1%)	8 (2.2%)		
	Unknown	4 (1.1%)	1 (0.3%)		
pathologic_stage, n (%)	Stage I	104 (28.5%)	66 (18.1%)	<0.001	Yates' correction
	Stage II	35 (9.6%)	49 (13.4%)		
	Stage III	29 (7.9%)	54 (14.8%)		
	Stage IV	2 (0.5%)	2 (0.5%)		
	Unknown	13 (3.6%)	11 (3%)		
T Stage, n (%)	T1	112 (30.7%)	68 (18.6%)	<0.001	Yates' correction
	T2	37 (10.1%)	54 (14.8%)		
	T3	28 (7.7%)	50 (13.7%)		
	T4	3 (0.8%)	10 (2.7%)		
	Tx	3 (0.8%)	0 (0%)		
M Stage, n (%)	M0	129 (35.3%)	134 (36.7%)	0.631	Yates' correction
	M1	1 (0.3%)	2 (0.5%)		
	MX	53 (14.5%)	46 (12.6%		
N Stage, n (%)	N0	120 (32.9%)	128 (35.1%)	0.615	Yates' correction
	N1	2 (0.5%)	2 (0.5%)		
	Nx	61 (16.7%)	52 (14.2%)		

**Figure 5:**
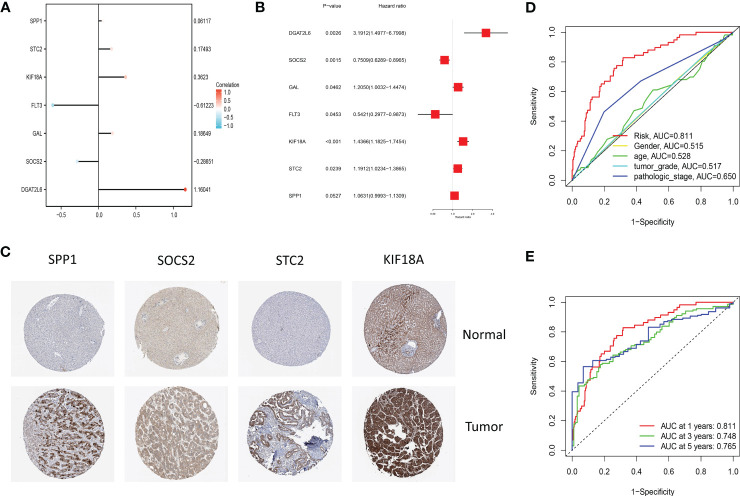
**(A)** The lollipop graph displays the variables and corresponding coefficients in the prognostic signature. **(B)** The forest plot shows the results of hazard ratios and 95% confidence intervals of signature genes from the multivariate Cox regression analysis. **(C)** Representative immunohistochemical staining images of SPP1 (antibody HPA074922, 10×), KIF18A (antibody HPA039312, 10×), SOCS2 (antibody CAB010356, 10×), and STC2 (antibody HPA045372, 10×) in normal and LIHC tissues are retrieved from The Human Protein Atlas database (HPA, https://www.proteinatlas.org/, accession date: January 2023). It should be noted that the immunohistochemistry staining of FLT3, GAL, and DGAT2L6 was absent from the HPA database. **(D)** The time-dependent ROC curves for different clinical characteristics in the TCGA cohort. **(E)** The time-dependent ROC curves for the prognostic signature in the TCGA cohort.

The proportion of patients who died was evidently higher in the high-risk group in contrast to low-risk group (**Figure 4E**). For evaluating the accuracy of prognostic characteristics predictions, we drew and compared the recipient operating characteristic and survival analysis curves. The findings revealed that the area under the ROC curve of the risk model was significantly larger compared to other clinical characteristics such as gender, age, tumor stage, and pathological stage (**Figure 5D**). And the area under the ROC curve was 0.811, 0.748 and 0.765 for OS at 1, 3 and 5 years in the TCGA cohort, separately (**Figure 5E**). Kaplan-Meier analysis demonstrated that the low-risk group of patients with LIHC had remarkably longer OS as compared to the high-risk group (**Figure 4F**).

Through plotting lollipop plots of variables (**Figure 5A**) as well as forest plots for multifactorial Cox regression analysis (**Figure 5B**), we found the prognostic signature of SOSC2 and FLT3 as prognostic protective factor for LIHC and other prognostic markers as risk factors. To further verify the signature gene expression patterns in LIHC patients, the expression profiles of proteins determined through immunohistochemical staining in HPA database were compared. The findings revealed that in contrast to the expression in normal tissues, four factors (KIF18A, SOCS2, SPP1 and STC2) in the prognostic signature were overexpressed in LIHC tissues (**Figure 5C**). The high expression of SOCS2 suggested a favorable prognosis for patients with LIHC.

### 3.4 Correlation analysis of prognostic characteristics of LIHC patients with clinical characteristics

**Figure 6:**
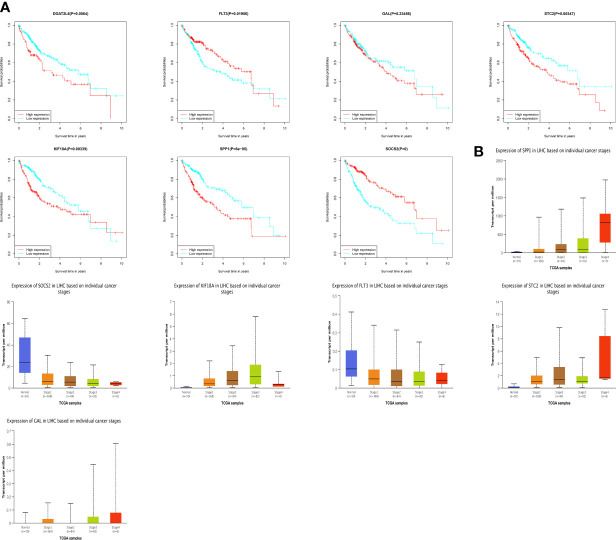
**(A)** Survival analysis of genes involved in the prognostic signature. **(B)** The genes involved in the prognostic signature expression among different tumor stages. DGAT2L6 was absent in the UALCAN database.

**Figure 7:**
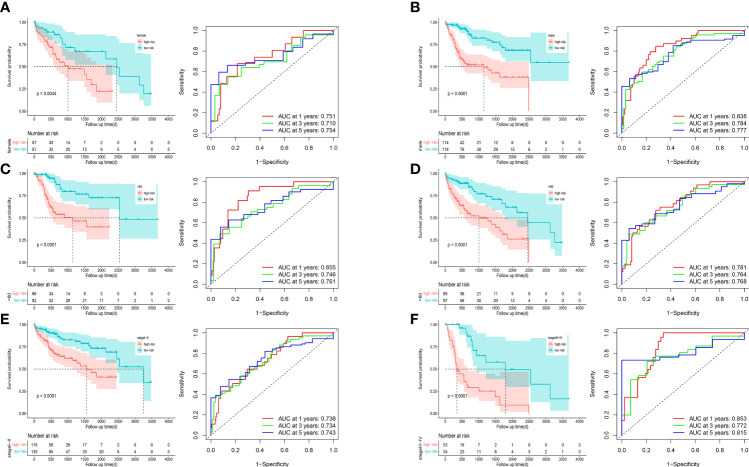
Survival curves and time-dependent ROC curves of patients with different gender **(A, B)**, ages **(C, D)**, and tumor stages **(E, F)** between high-risk and low-risk groups.

To understand the prognostic value and clinical relevance of prognostic characteristics in patients with LIHC, we first plotted survival curves to examine the predictive value of the genes implicated in the prognostic characteristics and, after log-rank testing, revealed that the high expression groups of SOSC2 and FLT3 had superior survival outcomes compared to low expression group, while the high expression groups of GAL, DGAT2L6, SPP1, STC2 and KIF18A had worse outcome of OS was worse in the high expression group in contrast to low expression group (**Figure 6A**). Subgroup analysis on the basis of clinical features revealed that the expression of risk factors DGAT2L6, GAL, KIF18A, STC2, and SPP1 in the prognostic signature was positively linked to the tumor stage (paradoxically, low expression of DGAT2L6 and KIF18A in tumor stage IV may be owing to the sample size being small). In contrast, the protective factors SOSC2 and FLT3 expression had a negative association with tumor stage (**Figure 6B**). To investigate the predictive power of risk model, a subgroup analysis was implemented on both risk groups of LIHC patients based on various clinical features. Comparable to the findings of training cohort, the survival rates of LIHC patients in group at high risk with varying clinical features were lower than those of patients in low-risk group (**Figure 7**). We identified that the risk model was a greater predictor of survival for men (**Figure 7B**), people under 60 years of age (**Figure 7C**) and advanced tumor patients (**Figure 7F**).

### 3.5 Construction of survival prognostic nomograms for LIHC and DCA evaluation

**Figure 8:**
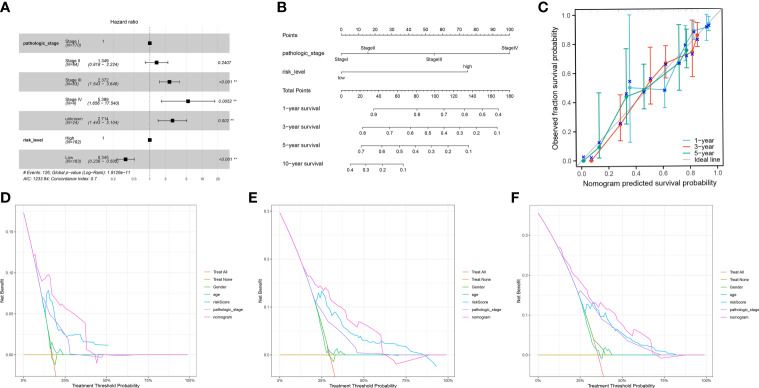
**(A)** The multivariate Cox regression model with clinical features included. **(B)** A nomogram model was constructed to predict the 1-year, 3-year, and 5-year overall survival of LIHC patients. **(C)** Calibration curves of the nomogram model for 1-year, 3-year, and 5-year overall survival. **(D-F)** Decision curve analysis for 1-year **(D)**, 3-year **(E)**, and 5-year **(F)** overall survival of the nomogram model.

We incorporated the clinical characteristics into the risk model and carried out the multivariate and univariate regression analyses (**Table 2**). The univariate Cox regression analysis suggested that sex, age, and the pathological stage were independent factors affecting prognosis, whereas multifactor regression analysis indicated that the risk score calculated based on the risk model together with tumor pathological stage were independent factors influencing prognosis (**Figure 8A**). Pathological stage was incorporated into the risk score model to establish the nomogram model (**Figure 8B**) for the prediction of OS at 1, 3, and 5 years (**Figure 7A**). Moreover, a calibration curve plot analysis of nomogram model was carried out and the calibration curve fitted well to the desired diagonal (**Figure 8C**). These outcomes suggest that the model has good discriminatory ability. Based on the DCA, the nomogram model predicted the OS of patients with liver cancer at 1, 3, and 5 years better than clinical characteristics such as TNM stage, age, and sex (**Figures 8D–F**).

**Table 2:** The univariate and multivariate Cox regression analyses of clinical characteristics for overall survival in LIHC patients.

Characteristics	Total(N)	Univariate analysis		Multivariate analysis	
		Hazard ratio (95% CI)	P value	Hazard ratio (95% CI)	P value
**Age**	365		0.196		
<65	216	Reference			
≥65	149	1.262 (0.888 - 1.794)	0.194		
**Gender**	365		0.367		
FEMALE	119	Reference			
MALE	246	0.845 (0.588 - 1.215)	0.364		
**Tumor_grade**	360		0.800		
G1	55	Reference			
G2	175	1.137 (0.670 - 1.930)	0.635		
G3	118	1.201 (0.689 - 2.094)	0.518		
G4	12	1.640 (0.606 - 4.442)	0.330		
**Pathological stage**	365		**< 0.001**		
Stage I	170	Reference		Reference	
Stage II	84	1.451 (0.881 - 2.390)	0.144	2.911 (0.602 - 14.085)	0.184
Stage III	83	2.700 (1.759 - 4.142)	**< 0.001**	1.544 (0.434 - 5.486)	0.502
Stage IV	4	5.645 (1.739 - 18.325)	**< 0.01**	0.000 (0.000 - Inf)	0.996
Unknown	24	2.680 (1.429 - 5.026)	**< 0.01**	1.933 (0.673 - 5.552)	0.221
**T Stage**	365		**< 0.001**		
T1	180	Reference		Reference	
T2	91	1.406 (0.876 - 2.258)	0.158	0.469 (0.105 - 2.100)	0.322
T3	78	2.603 (1.705 - 3.974)	**< 0.001**	1.636 (0.475 - 5.632)	0.435
T4	13	5.262 (2.626 - 10.547)	**< 0.001**	2.353 (0.680 - 8.141)	0.177
Tx	3	1.746 (0.240 - 12.720)	0.582	1.603 (0.187 - 13.734)	0.667
**M Stage**	365		**< 0.05**		
M0	263	Reference		Reference	
M1	3	4.149 (1.304 - 13.203)	**< 0.05**	27893.3627 (0.000 - Inf)	0.996
Mx	99	1.514 (1.032 - 2.220)	**< 0.05**	1.650 (1.061 - 2.568)	**< 0.05**
**N Stage**	365		0.200		
N0	248	Reference			
N1	4	2.051 (0.502 - 8.381)	0.317		
Nx	113	1.375 (0.941 - 2.009)	0.100		
**Risk score**	365		**< 0.001**		
High	182	Reference		Reference	
Low	183	0.328 (0.225 - 0.477)	**< 0.001**	0.330 (0.224 - 0.487)	**< 0.001**

The bold values denote statistical significance at P < 0.05 or P < 0.001.

### 3.6 Validation of the prognostic signature

**Figure 9:**
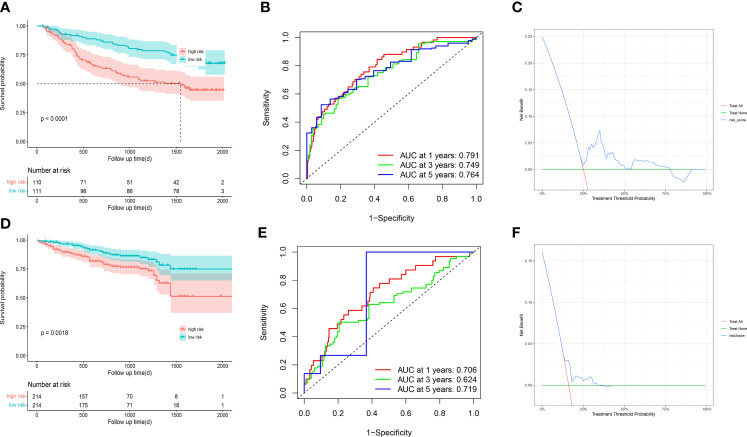
GSE14520 and ICGC-LIRI JP was used for validation **(A)** The overall survival curves of GSE14520 patients between high-risk and low-risk groups were plotted based on the prognostic signature. **(B)** The time-dependent ROC curves for different clinical characteristics in the GSE14520 cohort. **(C)** Decision curve analysis for 3-year overall survival of the prognostic model. **(D)** The overall survival curves of ICGC-LIRI JP patients between high-risk and low-risk groups were plotted based on the prognostic signature. **(E)** The time-dependent ROC curves for different clinical characteristics in the ICGC-LIRI JP cohort. **(F)** Decision curve analysis for 3-year overall survival of the prognostic model.

To further validate the stability and robustness of prognostic signature and its general applicability, we included GSE14520 and ICGC-LIRI JP as external validation cohorts. Prognostic marker gene expression was first analyzed in the dataset. We utilized a risk model to calculate risk scores and categorized patients into low- and high-risk groups in accordance with median scores. Survival curves (**Figures 9A, D**) and ROC curves over time (**Figures 9B, E**) were drawn, and proved to be markedly lower in the high-risk group versus low-risk group. The area under the ROC curve at 1, 3 and 5 years was 0.791, 0.749 and 0.75, separately, showing that the prognostic characteristics allow for greater differentiation between both risk groups. DCA of the model in GSE14520 and ICGC-LIRI JP (**Figures 9C, F**) showed better clinical benefits.

### 3.7 Functional enrichment analysis and mutation analysis

**Figure 10:**
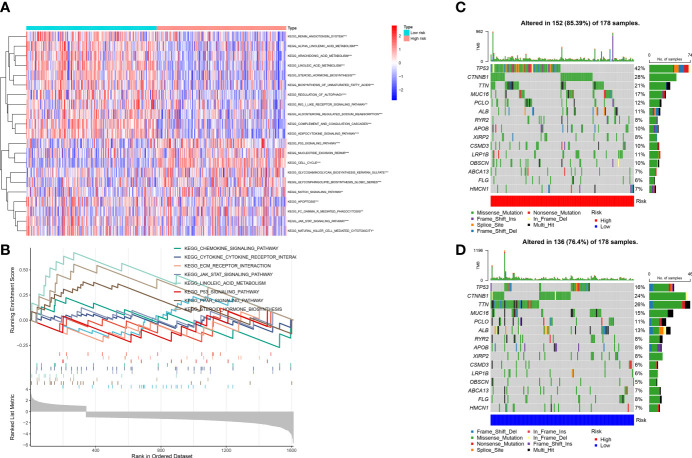
**(A)** Gene set variation analysis (GSVA) between high and low-risk groups. **(B)** Gene set enrichment analysis (GSEA) between high and low-risk groups. **(C, D)** Gene mutation analysis between high-risk and low-risk groups.

We conducted the GSVA and mutation analyses to get more insight into the mechanisms of survival differences. GSVA (**Figure 10A**) revealed that pathways such as pyrimidine metabolism, homologous recombination, and cell cycle were primarily enriched in high-risk groups. The pathways associated with lipid metabolism, for instance fatty acid metabolism and the adipocytokine signaling pathway; amino acid metabolism-related pathways, including the metabolism of glutamate, aspartate and alanine; the degradation of isoleucine, leucine and valine; the calcium signaling pathway; the biosynthesis of bile acids; as well as other lipid metabolic pathways, were enriched in low-risk group. Besides, GSEA showed that the pathways related to lipid metabolis, including steroid hormone synthesis, PPAR pathway, and linoleic acid metabolism, were predominantly enriched in low-risk group. Furthermore, the high-risk group was primarily enriched in pathways associated with immunity, including the chemokine signaling pathway, JAK-STAT pathway, the extracellular matrix receptor interactions and cytokine-cytokine-receptor interactions (**Figure 10B**). The above analysis suggested that there might be potential mechanisms at the mutation level in high-risk group. Therefore, we next conducted a mutational analysis of patients with LIHC (**Figures 10C, D**), where we first compared the genes with a high frequency of somatic mutations in LIHCs, like CTNNB1, TP53, MUC16 and TTN. The findings indicated that the mutation rates of CTNNB1 and TP53 were markedly higher in high-risk group, which could be a factor leading to the adverse prognosis of patients at high risk.

### 3.8 Immune infiltration analysis based on the prognostic signature

**Figure 11:**
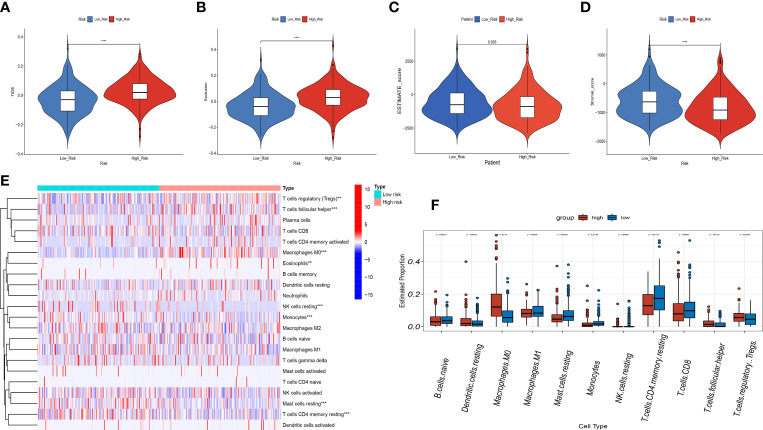
Correlation analysis of the risk score and immune infiltration in LIHC patients. **(A-D)** Comparison of the TIDE score **(A)**. exclusion score **(B)**. estimate score **(C)**. stromal score **(D)**. between the high-risk and low-risk groups. **(E)** The heatmap diagram displays the Immune infiltration difference between the high-risk and low-risk groups *via* Cibersort Algorithm. **(F)** The box diagram displays the Immune infiltration difference between the high-risk and low-risk groups *via* Cibersort Algorithm. **p < 0.01, and ***p < 0.001, ****p < 0.0001.

In view of the close connection between immune responses and prognostic features identified in the functional enrichment analysis, the relation between infiltrating immune cells and risk models was further investigated. We evaluated the differences in immune status among risk groups using the inverse convolution and CIBERSORT algorithms. In the immune cell type analysis, we detected a distinct difference in tumor immune infiltration between both risk groups. The high-risk group presented a greater abundance of M0-type macrophages, resting dendritic cells, regulatory T cells, and T-helper cell infiltration and a lower abundance of resting CD4 + T cells, CD8 + T cells, resting mast cells, naive B cells, and CD8+ T cell infiltration (**Figures 11E, F**). Associations between immune cells and prognostic features were examined with timer sites. The expression of KIF18A, SPP1, STC2 and SOCS2 presented a positive association with infiltration of dendritic cells and macrophages, while the expression of FLT3 presented a positive association with infiltration of CD8+ T cells and B cells (**Supplementary Figure 2**). High-risk group had a higher exclusion score and TIDE in contrast to low-risk group (**Figures 11A, B**). Additionally, the association between immune microenvironment scores and risk scores and stromal scores was statistically significant (**Figures 11C, D**).

### 3.9 Correlation of lipid metabolism and immune-related prognostic signature with single-cell properties

**Figure 12:**
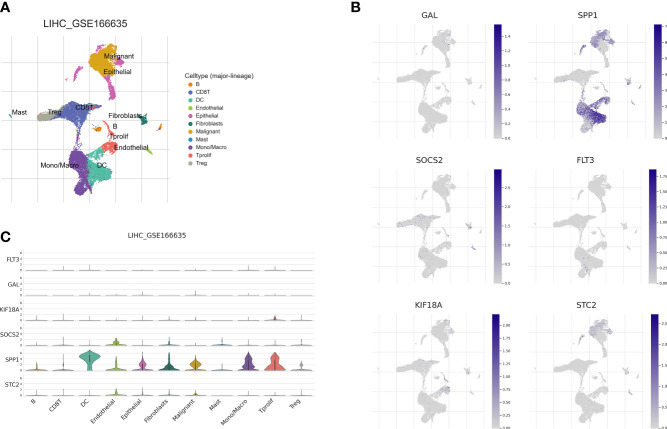
Correlation of the prognostic signature with single-cell clusters. **(A)** UMAP plot of ten major cell clusters in the LIHC tumor microenvironment. **(B)** The distribution of the prognostic genes in cell clusters. **(C)** Violin plot of the prognostic signature expression at the single-cell level.

Recently, single-cell sequencing has become an important tool for revealing cellular heterogeneity and differences. Further investigation of the action of prognostic genes in tumor microenvironment, we obtained GSE166635 data from the TISCH database and visualized UMAP in 10 cell clusters (**Figure 12A**), each of which was labeled according to its own characteristic genes as B, CD8T, DC, endothelial, epithelial fibroblasts, malignant, mast, mono/macro, and proliferative cell clusters. Based on the distribution of prognostic marker genes in the ten cell clusters (**Figure 12B**), SPP1 was primarily found in monocytes, DCs and malignant tumor cells. GAL was distributed in malignant cells. SOCS2 was distributed in endothelial cells, fibroblasts, and monocytes; FLT3 in monocytes; and STC2 in malignant cells. KIF18A was distributed in endothelial cells. To determine the expression characteristics of prognostic marker genes in immune microenvironment, we identified the genetic distribution in different cell clusters using violin plots (**Figure 12C**).

### 3.10 Screening of chemotherapeutic drugs versus small molecule drugs

**Figure 13:**
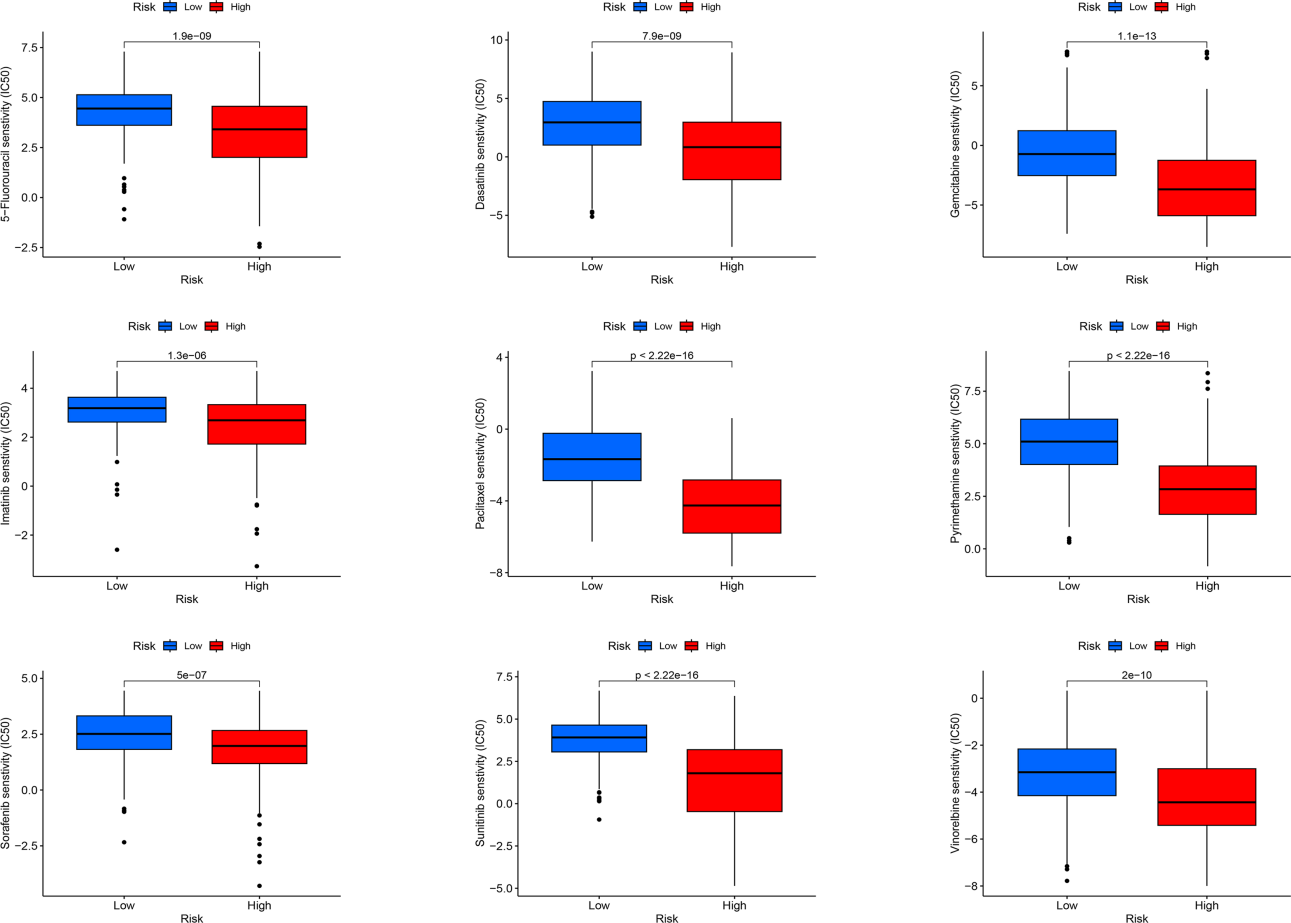
Sensitivity analysis of high-risk and low-risk patients to commonly used chemotherapy drugs.

**Figure 14:**
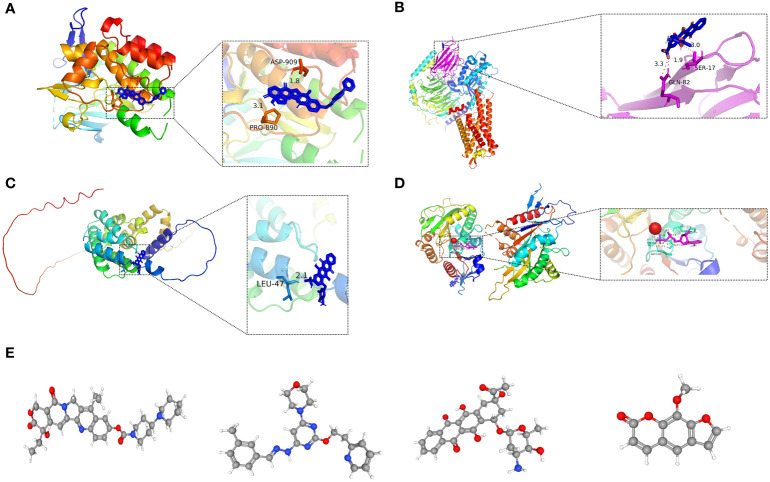
**(A-D)** Molecular docking pattern of key pharmacodynamic substances and core targets. **(A)** Irinotecan-SPP1. **(B)** Idarubicin-GAL. **(C)** Idarubicin -STC2. **(D)** Irinotecan-KIF18A.: **(E-H)** 3D structures of small molecule drugs predicted by the PubChem open chemical database,including irinotecan **(E)**, apilimod **(F)**, idarubicin **(G)**, and methoxsalen **(H)**.

For LIHC, we evaluated the capacity of risk models for predicting the effectiveness of commonly prescribed chemotherapeutic agents. **Figure 13** presents that low-risk group had higher half-maximal inhibitory concentration (IC50) values of fluorouracil, etanercept, sunitinib, paclitaxel, dasatinib, gemcitabine, imatinib, sorafenib, and vincristine in contrast to the high-risk group (p<0.05), suggesting that patients at high risk have greater sensitivity to chemotherapeutic agents and that these chemotherapeutic agents have greater clinical efficacy in patients at high risk. In summary, the findings revealed the potential predictive value of prognostic genes for chemotherapeutic efficacy in patients with LIHC. Additionally, we offloaded a list of DEGs between both risk groups. We predicted four small-molecule compounds that could be employed for LIHC treatment: idarubicin, irinotecan, methoxsalen and apilimod (**Figures 14E–H**).

### 3.11 Molecular docking

Molecular docking is an essential approach for drug design based on structure and for screening interacting molecules *via* the identification of optimal conformations of small-molecule targets and compounds (23). We molecularly docked four key targets (SPP1, GAL, KIF18A, and STC2) with their respective active small molecule compounds. Typically, the principles for investigating whether receptors and ligands can interact and their optimum binding mode are the complementarity of their spatial architectures and the energy minimization (24). **Figure 14** presents irinotecan formed hydrogen bonds with SPP1 at the ASP-909 and PRO-890 sites (**Figure 14A**), whereas it formed hydrogen bonds with KIF18A at the LYS-119, HIS-121, GLY-116, and GLY-11 sites (**Figure 14D**). Idarubicin formed hydrogen bonds through the GLN-82 site and SER-17 sites, interacting with GAL (**Figure 14B**). Idarubicin further formed hydrogen bonds through the LEU-47 site, interacting with STC2 (**Figure 14C**).

### 3.12 Expression of prognostic genes

**Figure 15:**
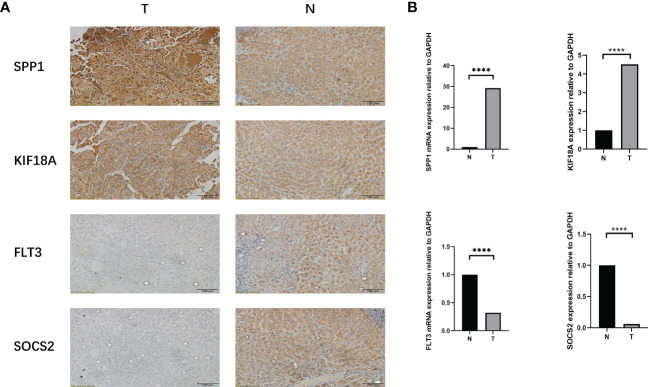
Expression of the prognostic genes in human. **(A)** IHC images of SPP1, KIF18A , FLT3 and SOCS2, in LIHC tissue and paracancerous tissue (magnification ×20). Scale bars: 100µm for 20×. N represents paracancerous tissues, and T represents LIHC tissues. **(B)** mRNA expression of SPP1, KIF18A , FLT3 and SOCS2 in LIHC tissues and paracancerous tissues. N represents paracancerous tissue, and T represents LIHC tissue. ****p< 0.0001.

To further verify the value of our prognostic model. We have selected several genes (SPP1, FLT3, KIF18A, SOCS2) of unclear significance in hepatocellular carcinoma. We investigated the expression of genes associated with prognosis in human tissues. In seven pairs of specimens from individuals with LIHC, qRT-PCR(**Figure 15B**) and IHC (**Figure 15A**) analysis revealed high SPP1 expression in tumor tissue, while FLT3, SOCS2 displayed the reverse trend. The findings revealed that FLT3, SOCS2 had high expression in paraneoplastic tissues, whereas SPP1, KIF18A were highly expressed in LIHC tissues. Taken together, lipid metabolism genes combined with immune-related genes are key to constructing a gene signature for patients with LIHC.

## 4 Discussion

Despite recent developments in neoadjuvant chemotherapy, molecularly targeted drugs, and immunotherapy, which have improved the efficacy of LIHC, the prognosis for long-term patient survival remains poor. Hence, more reliable and sensitive prognostic indicators are urgently required to monitor the progress of LIHC and to evaluate the survival of patients.

In the last few years, many researches have indicated that lipid metabolism in the tumor microenvironment modulates the invasion and proliferation of tumor cells and remodels the function of stromal cells, in particular immune cells, thus facilitating tumor metastasis (25). Hence, there is a strong association between anti-tumor immunity and the patterns of lipid metabolism. Nevertheless, to date, researchers have built prognostic models on the basis of a single lipid metabolism profile are either based on a single immune-related gene or have analyzed only the correlation between the model and immune environment of LIHC, and neither of them systematically combined the two for model construction, thus often suffering from poor validity, set robustness, and limitations of extrapolation. For example, Yan's study had a poor risk score correlation with immune cells (10), while another study lacked external set validation (11). Gu's report, on the other hand, lacked *in vitro* experimental validation (12), and each study possessed the drawback of not guaranteeing that the AUC values of the validation and training set risk models were still high. Aiming to overcome the deficiencies of previous research, this study integrated genes associated with lipid metabolism and immunity to enhance the robustness and accuracy of prognostic features through delivering multi-scale clinical characteristics.

First, data from LIHC patients were downloaded from TCGA database and examined with R software to get LRDGs. Subsequently, unsupervised consensus cluster-typing of LIHC was performed using LRDGs, and survival analysis showed differences in survival status among the different groups. Many immune-related pathways associated with LRDGs were enriched by GO analysis, which was performed according to ssGSEA for type I and differences in immune infiltration patterns were found among the different groups. These results confirmed that there exist many interactions between lipid metabolism and immunity during the development and progression of LIHC. Subsequently, we used LRDGs and IRDGs and acquired the prognostic characteristics of the seven genes *via* applying multivariate, univariate Cox regression analyses and LASSO. Of these, STC2, SPP1, FLT3 and GAL are found to be strongly linked to immunity and lipid metabolism. dGAT2L6, SOCS2, and KIF18A were associated with lipid metabolism. The model was used to score patients with LIHC, and survival analysis of the risk model presented that according to the Kaplan-Meier survival curve, the high-risk group had a marked shorter OS of patients with LIHC in contrast to the low-risk group (log-rank value< 0.001). ROC curve analysis over time showed that the predictive characteristics of LIHC were more accurate in the prediction of survival. In addition, the external validation results based on the GSE14520 and ICGC-LIRI JP datasets confirmed the robustness of the predictive features relative to previous studies.

SPP1 influences the malignant biological activity and immune escape of tumor cells and its overexpression facilitates the progression and metastasis of LIHC (26, 27). SPP1 was previously considered a potential marker for early recurrence and poor prognosis of LIHC and a major metastasis-related gene (28, 29). A meta-analysis of seven researches demonstrated that raised levels of plasma SPP1 have comparable diagnostic performance to AFP-based results (30, 31). However, elevated SPP1 levels may be associated with other malignancies and should therefore be combined with other LIHC-specific biomarkers (32). Patients with LIHC and high STC2 expression have poor prognoses, and STC2 promotes local angiogenesis, tumor proliferation, and metastasis (33, 34). As a member of the SOCS family, the suppressor of cytokine signaling 2 (SOCS2) is present in numerous types of tumor progression. SOCS2 overexpression reduced the ability of LIHC cells to migrate and invade *in vitro*, and suppressed their metastasis *in vivo* (35). SOCS2 deficiency facilitates spontaneous progression of intestinal tumors that are driven both by AP-1 activation and mutations in the *E. coli*/β-catenin pathway (36). KIF18A mediates organelle and protein transport and plays a role in microtubule motility during cytokinesis and mitotic chromosome arrangement (37). KIF18A is also associated with metastasis of solid tumors (e.g., breast cancer (38)). Specific kinesins and molecules involved in the cell cycle are potential targets (39). FLT3 targets sorafenib and is closely linked to the efficacy and patient survival of sorafenib. Patients with LIHC stratified on the basis of high levels of FLT3 may gain from treatment with sorafenib (40). Nevertheless, no literature has proven that high levels of FLT3 are related to a better prognosis in LIHC patients. This study raises the following relevant question: This study found that although previous studies have shown that glycopeptides and GMAP pro-peptides (GAL) are activated in human LIHC and tend to accumulate in the stromal tissue surrounding LIHC cells (41), DGAT2L6 did not correlate with LIHC and could be used as a potential prognostic marker.

Subgroup analysis on the basis of clinical features showed good agreement between prognostic indicators and disease stage. The inclusion of clinical characteristics in the multivariate and univariate Cox regression analyses suggested that age, gender, risk score and pathological stage can be considered as independent prognostic factors. The risk score and pathological staging can be treated as independent prognostic factors in a multifactorial analysis. On this basis, a model of prognostic nomogram was constructed, and a column score plot, calibration curve, and clinical decision curve were developed. The calibration curve displayed the confidence of the model, and DCA exhibited the clinical application of this prognostic model. For further examination of the basic mechanisms influencing survival differences, we originally compared genes with a high somatic mutation frequency in LIHC, which include CTNNB1, TP53, MUC16 and TTN between groups. The findings of the study suggested that the mutation rates of CTNNB1 and TP53 were closely related to the risk score, which may result in a poor prognosis for the high-risk group.

To further elucidate the underlying mechanisms associated with immune affecting the prognosis of patients with LIHC, we used an inverse convolution algorithm, CIBERSORT. The TIDE score is derived from cytotoxic T lymphocyte function, which has a negative relation with clinical response to OS and immune checkpoint blockade (ICB) (18). Both TIDE algorithms (TIDE score and exclusion score) suggested relatively low sensitivity to immune checkpoint suppressors in high-risk group of patients with LIHC. Besides, the XCell algorithm-based interstitial and microenvironment scores showed higher LIHC interstitial and microenvironment scores in the low-risk group, which confirmed the low immunogenicity and responsiveness of tumors to ICBs. CIBERSORT algorithm was applied for quantifying the function and infiltration of immune cells and we revealed that the high-risk group had more resting dendritic cells, regulatory T cells, M0 macrophages, T helper cells and Treg cells, and fewer resting CD4+ T cells, CD8+ T cells, resting mast cells, CD8+ T cells and initial B cells as compared to low-risk group. Th1-type immune responses that are activated from antigen presentation are a critical component of the antitumor action of M1 macrophages. Increased concentrations of M1 macrophages secrete multiple inflammatory factors to maintain the long-term inflammatory environment and enroll and initiate T cells in the early stages of tumors (42). In contrast, M2 macrophages that are activated by IL-13 and IL-4 are often employed as accelerators of tumor progression. They reduce the immune response and contribute to inflammation through the secretion of the suppressive cytokines TGF-β or IL-10 (43). They also secrete MMPs, which assist tumor cells to break through the endothelial cell basal layer and achieve metastasis (44). As resting macrophages, macrophages M0 are prone to convert to M2-like subtypes in tumor microenvironment (45). IFN-γ, LPS, or GM-CSF can induce M1-type macrophages. M1-type macrophages promote the inflammatory response and kill intracellular pathogens in tumors by releasing inflammatory mediators such as IL-1. M2 macrophages, which are induced with IL-13 and IL-4, highly express CD206, increase endocytosis and secrete the anti-inflammatory cytokines, for instance TGF-β and IL-10, facilitate Th2 cell differentiation, and participate in immune regulation, repair function, wound healing, angiogenesis, and promote tumor progression. In addition, the resting-state DC infiltration was higher in the high-risk group. DCs act a critical player in activating anti-tumor-associated T cells as specific antigen-presenting cells (46). The lack of DC activation was responsible for poor prognosis in the high-risk group. It is notable that the low-risk group has an evident higher level of mast cell infiltration. Previous researches have suggested that mast cells are an essential source of VEGF, which facilitates the proliferation and angiogenesis of tumors (47, 48). Nevertheless, recent studies have characterized the heterogeneity of mast cells and proved that the subpopulation of CD103+ mast cells display a stronger expression of molecules associated with antigen presentation, which include CD80, ICAM-1 as well as MHC-II-like molecules, which effectively activate CD4+ T cells in turn (49). The proportion of CD4 memory resting T cell infiltration decreased significantly with increasing patient risk. Similar to our findings, a related study reported that exacerbated infiltration of CD4 memory T cells occurred at the tumor sites (50). In tumor immunotherapy, the synergistic effect of CD4+ T and NK cells is stronger than that of CD8+ T cells (51). CD8+ T cells are activated *via* the recognition of tumor antigens through the T cell receptor (TCR) and rapidly proliferate and differentiate into the cytotoxic T cells, resulting in the elimination of tumor cells *via* cell-cell contact, which accounts for the higher infiltration levels of CD8+ T cells in low-risk group. Conversely, the infiltration levels of Treg cell were higher in high-risk group, and the rise in Treg cells and their synergy with other immune cells sustained immune tolerance of tumor cells, indicating that the infiltration of Treg cells in the tumor microenvironment is strongly linked to poor prognosis and that clearance of Treg cells may activate and strengthen the anti-tumor immune response (52). Researches have indicated that Treg cells exert an essential role in the microenvironment, prognosis as well as response to chemotherapy in various tumors (53), and enhanced infiltration density of FoxP3 regulatory T cells is closely linked to poor prognosis in a number of tumors, for instance melanoma, lung cancer, cervical cancer, gastric cancer and liver cancer (54). This may be the reason why a higher number of Treg in high-risk group in our research was related to a poorer prognosis.

Chemotherapy is currently the most widespread and effective tumor treatment, serving an essential role in killing tumor cells, suppressing tumor growth, and prolonging the survival of patients (55). Nevertheless, the emergence of chemoresistance in tumor patients presents a huge challenge to the treatment of cancer. Therefore, it is clinically important to investigate the mechanisms of drug resistance and enhance sensitivity to chemotherapy (56). High-risk group had evidently lower IC50 values of the chemotherapeutic agents fluorouracil, etanercept, sunitinib, paclitaxel, dasatinib, gemcitabine, imatinib, sorafenib, and vincristine than low-risk group, displaying that patients at high risk may gain more benefit from chemotherapy with this class of drugs. Drug prediction and molecular docking were then conducted, showing that all four drugs bound better to proteins encoded by poor prognostic target genes.

## 5 Conclusion

In conclusion, we first developed and validate a new prognostic signature based on genes related to immune and lipid metabolism in LIHC patients. We show here its robust performance in predicting prognosis, infiltration of immune cells as well as response to chemotherapy in LIHC. Furthermore, our study predicted possible drugs, as a groundwork for future developments. The use of dual signatures to predict small-molecule drug efficacy new method for the pharmacological treatment of patients. Our findings will further help predict OS and treatment effects of chemotherapy and immune checkpoint inhibition.

## Data availability statement

The original contributions presented in the study are included in the article/**Supplementary Material**. Further inquiries can be directed to the corresponding author.

## Ethics statement

The studies involving human participants were reviewed and approved by The Medical Research Ethics Committee of Guangdong Second People's Hospital. The patients/participants provided their written informed consent to participate in this study. Written informed consent was obtained from the individual(s) for the publication of any potentially identifiable images or data included in this article.

## Author contributions

TY: Writing the article, data collection, conception and design. YL: Conception and design, analysis and interpretation. JL: Writing the article, critical revision of the article, methodology. FL: Formal analysis, critical revision of the article. ZM, GL, HL: Critical revision of the article. JW, CC, XZ: Conception and design, critical revision of the article. All authors contributed to the article and approved the submitted version.

## Supplementary material

Supplementary Figure 1 Validation of the unsupervised consensus clustering patterns in the external five GEO datasets. **(A)** unsupervised consensus clustering heatmap. **(B)** The plot of the relative area changes from k=2 to 9 under the cumulative distribution function (CDF) curve. **(C)** Consistent CDF plot. **(D)** Tracing plot of clustered samples

Supplementary Figure 2 Enrichment analysis of IRDGs and LRDGs. **(A)** Go enrichment results circle plot of IRDGs and LRDGs. **(B)** Display of BP,MF,CC results in GO enrichment. **(C, D)** KEGG analysis of IRDGs and LRDGs.

Supplementary Figure 3 Tracking tumor immunophenotype(TIP) analysis of high and low risk groups. There are significant differcences between groups during step4 MDSC.recruiting and step5

Supplementary Figure 4 Analysis of the correlation between prognostic genes and immune cells. **(A)** KIF18A correlates with immune cell infiltration. **(B)** SOCS2 correlates with immune cell infiltration. **(C)** SPP1 correlates with immune cell infiltration. **(D)** STC2 correlates with immune cell infiltration. **(E)** FLT3 correlates with immune cell infiltration. **(F)** GAL correlates with immune cell infiltration. **(G)** DAGT2L6 correlates with immune cell infiltration.
