# Supplementary figures and images for: A novel signature incorporating lipid metabolism- and immune-related genes to predict the prognosis and immune landscape in hepatocellular carcinoma
Source: Front Oncol. 2023 Jun 6;13:1182434. doi: 10.3389/fonc.2023.1182434 (PMC10279962; doi:10.3389/fonc.2023.1182434)

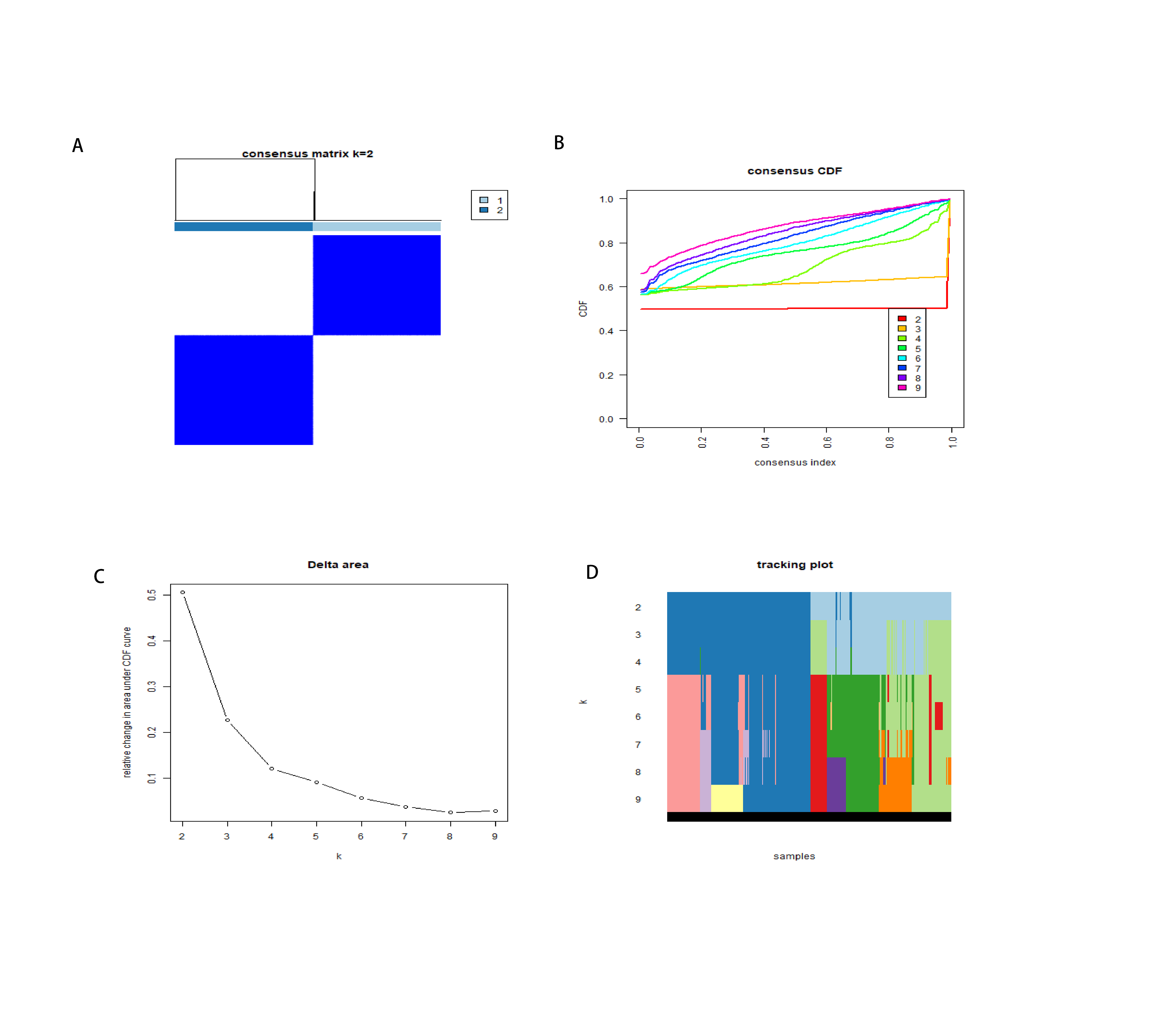

Supplement: Supplementary Figure 1 — Validation of the unsupervised consensus clustering patterns in the external five GEO datasets. (A) unsupervised consensus clustering heatmap. (B) The plot of the relative area changes from k=2 to 9 under the cumulative distribution function (CDF) curve. (C) Consistent CDF plot. (D) Tracing plot of clustered samples [file Image_1.tif]

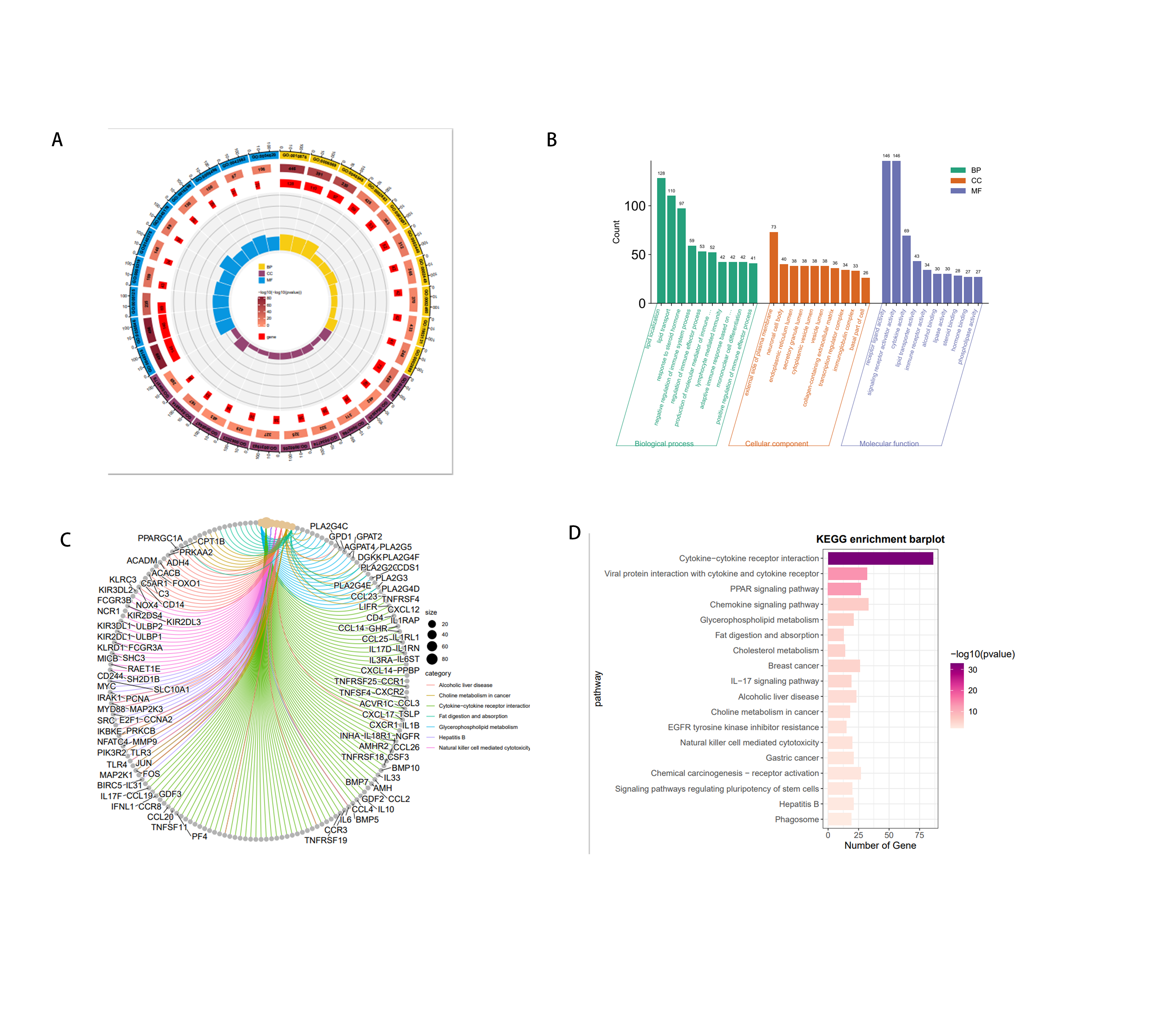

Supplement: Supplementary Figure 2 — Enrichment analysis of IRDGs and LRDGs. (A) Go enrichment results circle plot of IRDGs and LRDGs. (B) Display of BP,MF,CC results in GO enrichment. (C, D) KEGG analysis of IRDGs and LRDGs. [file Image_2.tif]

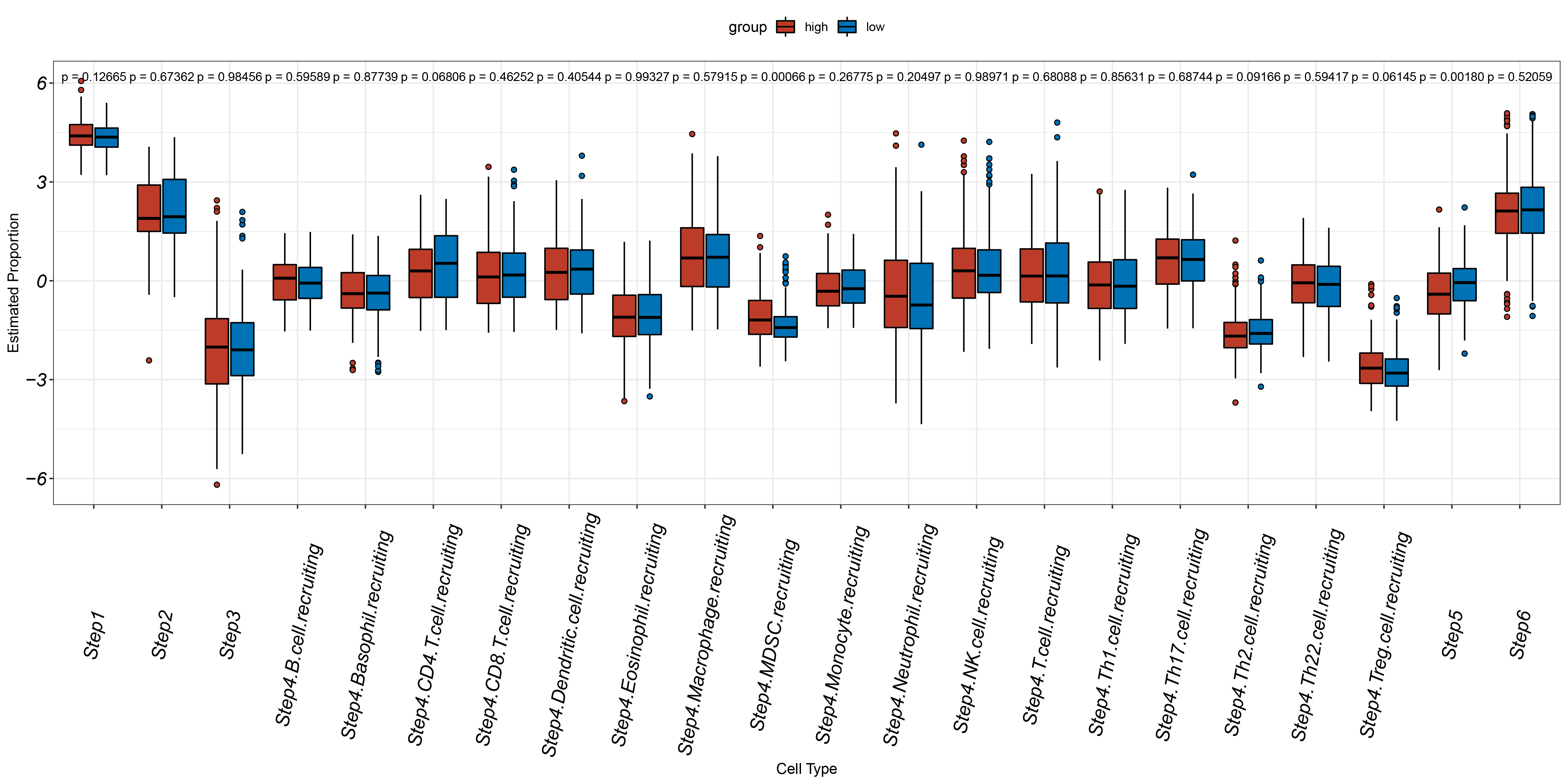

Supplement: Supplementary Figure 3 — Tracking tumor immunophenotype(TIP) analysis of high and low risk groups. There are significant differcences between groups during step4 MDSC.recruiting and step5 [file Image_3.tif]

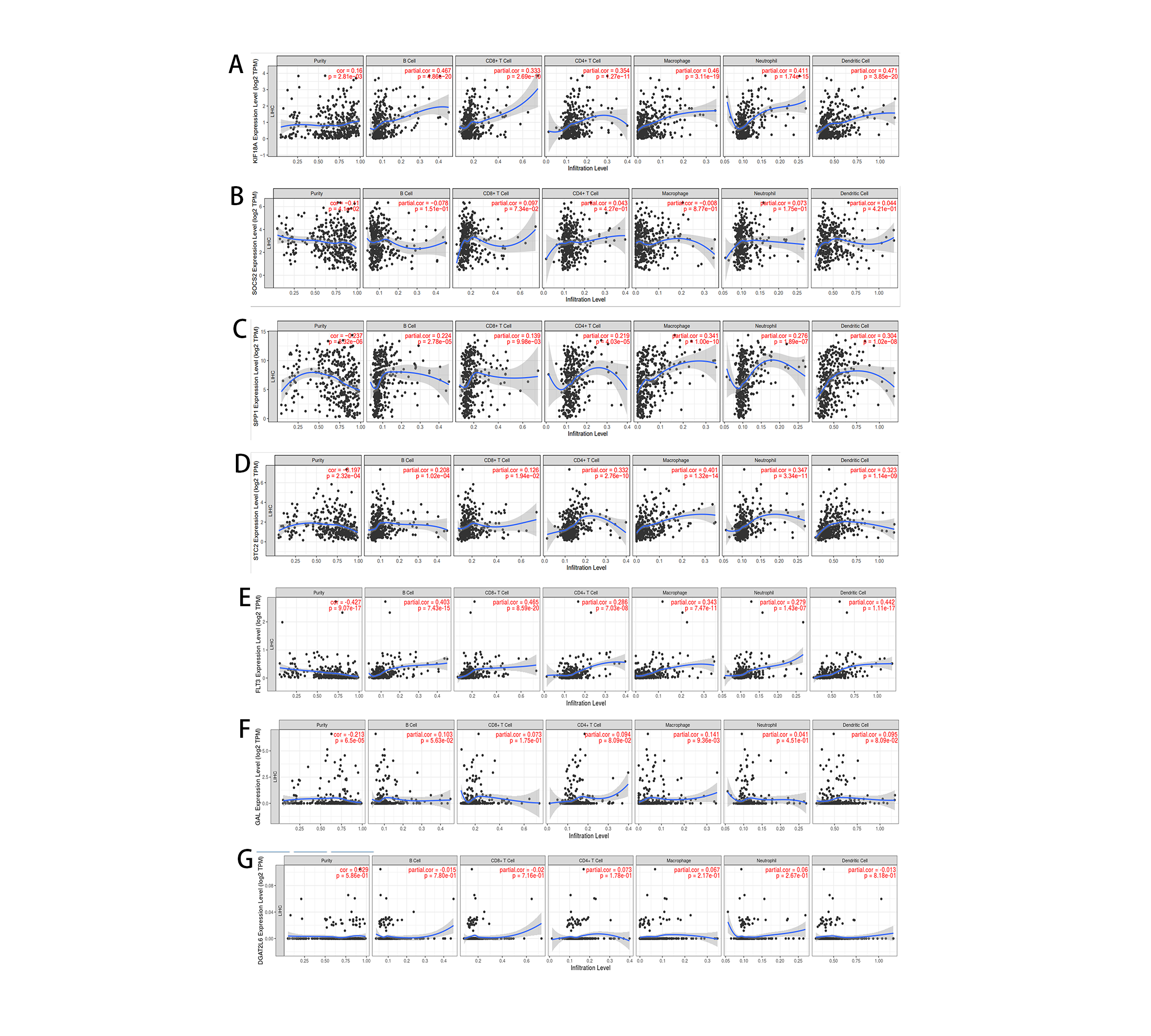

Supplement: Supplementary Figure 4 — Analysis of the correlation between prognostic genes and immune cells. (A) KIF18A correlates with immune cell infiltration. (B) SOCS2 correlates with immune cell infiltration. (C) SPP1 correlates with immune cell infiltration. (D) STC2 correlates with immune cell infiltration. (E) FLT3 correlates with immune cell infiltration. (F) GAL correlates with immune cell infiltration. (G) DAGT2L6 correlates with immune cell infiltration. [file Image_4.tif]
